# Supplementary material for: Supplements and Nutritional Interventions to Augment High-Intensity Interval Training Physiological and Performance Adaptations—A Narrative Review
Source: Nutrients. 2020 Jan 31;12(2):390. doi: 10.3390/nu12020390 (PMC7071320; doi:10.3390/nu12020390)
Supplement: Supplementary file 1 [file nutrients-12-00390-s001.pdf]

**Table S1:** Research to date involving the delivery of dietary inorganic nitrate supplementation and high-intensity, aerobic-type exercise.

| Study | Participants | Exercise | Timing of Supplementation | Supplementation | Results |
|---|---|---|---|---|---|
| **Repeat Sprints** | | | | | |
| Kent et al. 2018 | Trained, team-sport athletes (male; n=12) | Cycling, repeat sprints (9 x 4s) at sea level or 3000 meter simulated altitude | 120 min prior to exercise | 13.0 mmol $NO_3^-$ delivered as beet root juice | No effect on peak or mean power output; reduced V02 during submaximal warm-up |
| Muggeridge et al. 2017 | Trained male kayakers (male; n=8) | Kayak ergometer repeat sprints 5 x 10 sec, 50 sec of rest | 180 min prior to exercise | 5.0 mmol $NO_3^-$ delivered as beet root juice | Increase average power output |
| Aucouturier et al. 2015 | Recreationally trained, team sport players (male; n=12) | Cycle ergometer repeat 15 sec sprints with 30 sec rest until exhaustion; 170% of maximum aerobic capacity | 180 min prior to exercise | 10.9 mmol $NO_3^-$ delivered as beet root juice | Increase repetitions and total workload |
| Buck et al. 2015 | Amateur, team sport players (female, n=13) | 6 x 20 meter, repeated sprints performed 3 times (start, half-time, and end) of a simulated team game circuit | 3 hours prior to exercise | 6.0 mmol $NO_3^-$ delivered as beet root juice (placebo added) | No improvements in total sprint times |
| Thompson et al. 2017 | Recreationally trained, team sports players (male; n=16) | 2 sets of cycle ergometer sprints; 5 sets of 6 sec sprints with 14 sec rest ; occurred MID and POST- | 7 days | 12.8 mmol $NO_3^-$ delivered as beet root juice | Work volume increase at MID & work volume increase at POST; |

| | | | | | |
|---|---|---|---|---|---|
| | | simulated team-sport matches | | | increase in work volume for entire session |
| Clifford et al. 2016 | Competition trained, team sport players (male, n=20) | 2 sets of RST; 20 x 30 m sprints w/ 30 sec rest | 150 min prior | 11.4 mmol $NO_3^-$ delivered as beet root juice | No effect on mean or average sprint times |
| Martin et al. 2014 | Recreationally-trained, team sport players (male; n=16) | Cycle ergometer repeat 8 sec sprints with 30 sec rest until exhaustion | 120 min prior to exercise | 6.4 mmol $NO_3^-$ delivered as beet root juice | Decreased total number of repetitions and decreased total work |
| Wylie et al. 2013 | Recreationally trained, team sport players (male, n=10) | Cycle ergometer, repeat sprints;<br><br>1:4 ratio<br>24 x [6 sec sprint; 24 sec rest]<br><br>1:8 ratio<br>7 x [30 sec sprint; 4 min rest]<br><br>1:1 ratio<br>6 x [60 sec sprint x 60 sec rest] | 5 days | 8.4 mmol $NO_3^-$ delivered as beet root juice | Improved mean average power and improved mean peak power for 1:4 ratio sprints |
